# The Live Long Walk Strong Rehabilitation Program: What Features Improve Mobility Skills? Virtual Pilot

**DOI:** 10.1093/geroni/igab046.3447

**Published:** 2021-12-17

**Authors:** Rebekah Harris, Jonathan Bean, Elisa Ogawa, Addie Middleton, Catherine Kelly

**Affiliations:** 1 VA Boston Healthcare System, VA Boston Healthcare System, Massachusetts, United States; 2 VA Boston, Boston, Massachusetts, United States

## Abstract

To evaluate the feasibility of delivering the Live Long Walk Strong (LLWS) rehabilitation program among community dwelling, mobility limited older Veterans in the VA Boston Healthcare System. Community dwelling Veterans 50 years and older identified as being at high risk for mobility decline based on self-report task modification and AM-PAC mobility questions. All Veterans received 10 sessions over 8 weeks of LLWS Physical Therapy care focusing on novel impairments related to mobility decline and behavioral change strategies. Sessions were delivered 1:1 with a Physical Therapist over 45 minutes. To assess feasibility, we tracked recruitment and retention metrics. We assessed length of each session, number of sessions attended, and any reason for withdrawal. To examine technological feasibility, we recorded number and type of issue along with resolution of the issue. A total of 178 Veterans were contacted to participate. Twenty Veterans were enrolled into the LLWS virtual pilot between October 2020 – May 2021. Among our 20 enrolled, 5 did not complete the program. Reasons for not completing included: being enrolled in another exercise study simultaneously and non-related medical complications. Among those completing, an average of 9.7 out of 10 intervention sessions were completed. An average of 1.8 technology difficulties per Veteran was experienced within the intervention. The most frequent technology issues experienced were related to camera positioning and Wi-Fi bandwidth resulting in delayed video and audio. LLWS is feasible to deliver as a virtual mode of care in middle and older aged Veterans at high risk for mobility decline.

